# Direct comparison of shot-to-shot noise performance of all normal dispersion and anomalous dispersion supercontinuum pumped with sub-picosecond pulse fiber-based laser

**DOI:** 10.1038/srep19284

**Published:** 2016-01-13

**Authors:** Mariusz Klimczak, Grzegorz Soboń, Rafał Kasztelanic, Krzysztof M. Abramski, Ryszard Buczyński

**Affiliations:** 1 Glass Department, Institute of Electronic Materials Technology, Wolczynska 133, 01-919 Warsaw, Poland; 2 Laser & Fiber Electronics Group, Wroclaw University of Technology, Wybrzeze Wyspianskiego 27, 50-370 Wroclaw, Poland; 3 Faculty of Physics, University of Warsaw, Pasteura 7, 02-093 Warsaw, Poland

## Abstract

Coherence of supercontinuum sources is critical for applications involving characterization of ultrafast or rarely occurring phenomena. With the demonstrated spectral coverage of supercontinuum extending from near-infrared to over 10 μm in a single nonlinear fiber, there has been a clear push for the bandwidth rather than for attempting to optimize the dynamic properties of the generated spectrum. In this work we provide an experimental assessment of the shot-to-shot noise performance of supercontinuum generation in two types of soft glass photonic crystal fibers. Phase coherence and intensity fluctuations are compared for the cases of an anomalous dispersion-pumped fiber and an all-normal dispersion fiber. With the use of the dispersive Fourier transformation method, we demonstrate that a factor of 100 improvement in signal-to-noise ratio is achieved in the normal-dispersion over anomalous dispersion-pumped fiber for 390 fs long pump pulses. A double-clad design of the photonic lattice of the fiber is further postulated to enable a pump-related seeding mechanism of normal-dispersion supercontinuum broadening under sub-picosecond pumping, which is otherwise known for similar noise characteristics as modulation instability driven, soliton-based spectra.

Supercontinuum sources (SC) find increasingly widespread use in disciplines relying on high precision optical characterization, including optical coherence tomography^1^, spectroscopy^2^ or microscopy^3^. Many of these applications, which are related to biotechnology or medicine^4^, would still benefit from improvement of the coherence of the generated broadband radiation. Octave-spanning supercontinuum generation is required for absolute stabilization of self-referenced frequency combs, through the f-2f interference technique^5^,^6^. For this application, a low-noise, stable and coherent supercontinuum is particularly desirable. It has also been proven that ultrashort pulse broadening in all-normal dispersion photonic crystal fibers (PCFs) allows the limits in generation of few-cycle pulses from conventional anomalous dispersion fibers^7^ to be overcome. Mastering of glass synthesis and optical fiber drawing technologies has led to the demonstration of new, highly nonlinear chalcogenide optical fibers and of supercontinuum spectra covering wavelengths even up to 13.3 μm^8^. SC generation up to around 4–5 μm was reported earlier in fluoride, tellurite and chalcogenide step-index fibers or PCFs^9^. The most efficient spectral broadening, observable under pumping into anomalous dispersion wavelengths of the nonlinear fiber, is also characterized by large shot-to-shot variability, due to the stochastic nature of modulation instability (MI), whenever this process dominates the spectral broadening^10^. Different approaches to stabilization of anomalous dispersion-pumped SC have been reported. Femtosecond laser pumping is considered the most straightforward method^11^, since then in the anomalous dispersion soliton fission (SF) and soliton self-frequency shift dominate the broadening, while the contribution of the MI compared to picosecond and nanosecond pumping is strongly suppressed. More elaborate solutions are based on seeding, which introduces a seed wavelength component with deterministic phase in a four-wave mixing process^12^,^13^,^14^. An alternative approach makes use of fiber tapers, in which initial broadening occurs in the anomalous dispersion-pumped regime, and evolves into normal dispersion broadening during propagation along the taper^15^. Numerical results have also been reported predicting shot-to-shot noise control by self-similar compression in fiber tapers with only slightly varying dispersion^16^. In optical fiber-based SC generation, yet another alternative is to contain the spectrum entirely within the normal dispersion range of wavelengths (ANDi SC). In combination with femtosecond pulse pumping (less than 200 fs), coherent spectra spanning a full octave have been reported^17^,^18^,^19^,^20^. With pump pulse durations exceeding roughly 200–300 fs, the excellent coherence properties of ANDi SC are lost, due to Raman scattering^21^. Fiber birefringence has also been shown to have a detrimental effect on the stability of ANDi broadening^22^,^23^. We recently reported a highly coherent ANDi SC generation with 390 fs pump pulses^24^. In this case the spectrum was stabilized by seeding from part of nonlinearly unconverted pump radiation co-propagating in the photonic cladding of an all-solid glass ANDi photonic crystal fiber.

Different approaches to the characterization of SC phase coherence and intensity fluctuations have been reported. Fluctuations of phase can be characterized with the modulus of the complex first-order degree of coherence^10^. It can be experimentally obtained in an interferometric measurement from the contrast of the fringe pattern^25^. An extended approach to obtaining of SC coherence time and bandwidth has also been proposed on the basis of the second order coherence theory^26^. An enhanced FROG-based approach with improved temporal resolution and broad bandwidth has also been demonstrated^27^. Resolving of the individual ultrafast SC spectra can be done by the dispersive Fourier transformation method (DFT)^28^, albeit it is a phase insensitive measurement. It allows however to analyze the intensity noise performance of ultrafast sources in real-time and to compute wavelength correlation maps^29^,^30^. The DFT techniques enables to overcome the limitation stemming from ensemble averaging of available optical spectrum analyzers, while the entire experimental setup remains relatively simple. It is to be noted that characterization of nanosecond scale pulses has been reported using direct measurement with a fast photodiode^31^.

In this work we report the direct comparison of shot-to-shot noise performance of all-normal dispersion and anomalous dispersion-pumped supercontinuum generation. All-solid soft glass normal dispersion and regular lattice air-hole soft glass photonic crystal fibers (PCF) are used as the nonlinear media and an erbium fiber-based chirped pulse amplification system delivering 390 fs/1560 nm pulses as the pump source. The phase coherence of the SC was assessed by measuring the fringe visibility in an unequal path, Michelson-type fiber interferometer, similarly to previous works^25^,^32^. Real-time resolving of the spectra of several-picosecond scale output pulses of supercontinuum was done using the DFT method. Spectral correlation maps are plotted with a double purpose in mind. Firstly, by revealing the well-known physics behind soliton-based broadening, they support the use of the anomalous dispersion PCF as a general demonstrator. Secondly, the mechanism of shot-to-shot intensity stabilization of ANDi SC in the presence of Raman scattering is discussed and related to the mode structure of the demonstrated ANDi PCF. We suggest a new mechanism for spectral stabilization of Raman-scattering based, sub-picosecond pumped SC generation. Furthermore, with the use of spectral correlation maps we explain its pump power-dependent evolution. In the final part, we use single mode and multi-mode nonlinear simulations of pulse propagation to confront the cases of ANDi SC generation with and without the seeding mechanism included in the simulation.

Our results are of importance for supercontinuum source device engineering. They demonstrate that pumping of ANDi SC with sub-picosecond fiber lasers can be considered without any of the coherence trade-offs identified for earlier types of normal dispersion PCFs pumped in similar conditions^21^. Such pump lasers are cheaper, more robust and deliver more power, than the usually more complex and more expensive femtosecond systems. The seeding mechanism of stabilization, coming directly from the pump laser, is also of great convenience as it automatically fulfills the requirement for coherence between the pump and the seed.

## Discussion of the phase coherence of supercontinuum

**Figure 1:**
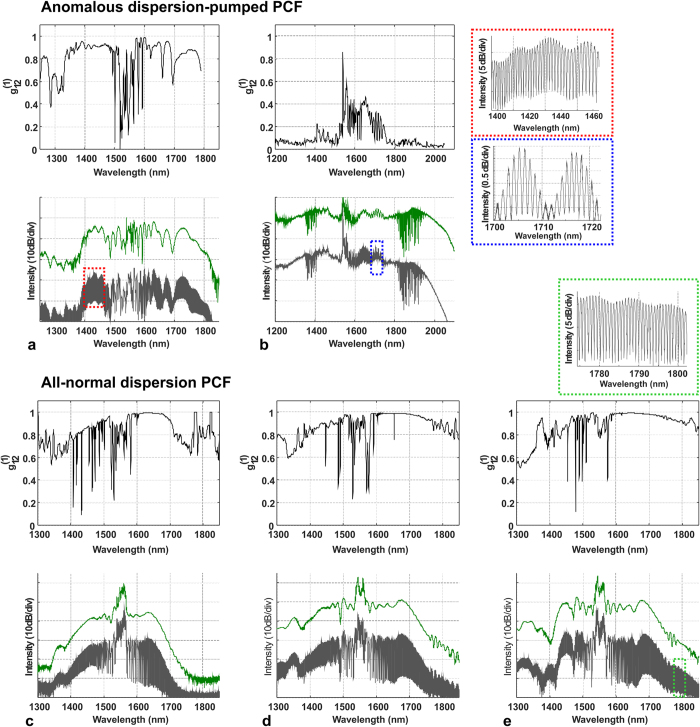
Results of interference measurements of SC spectra and profiles of spectral degree of coherence (**a**) anomalous dispersion-pumped PCF, coupled pump pulse energy E_in_ = 2 nJ, detail of interference fringes shown in red dotted box (**b**) anomalous dispersion-pumped PCF, E_in_ = 5 nJ, fringe detail shown in blue dotted box, (**c**) ANDi PCF, E_in_ = 2 nJ, (**d**) ANDi PCF, E_in_ = 3 nJ, (**e**) ADNi PCF, E_in_ = 5 nJ, fringe detail shown in green dotted box.

The SC signals, generated in each of the fibers were first launched into an unequal path Michelson-type interferometer in order to investigate the phase coherence properties for the two cases (details on the interferometer setup are provided in the Methods section). The interference patterns of the SC spectra were recorded for different pump power levels, corresponding to various stages of the development of spectra. The modulus of the complex degree of coherence, calculated as the contrast of the interference fringes (see Methods section) is shown in Fig. 1, along with the corresponding SC spectra and interferograms. Incident pump power was set such, that the output SC average power changed between about 150 mW and 450 mW. This corresponded to pump pulse energies of roughly 2–5 nJ and allowed to maintain the spectral width in a range which could be characterized in the available DFT setup in the next section. Figure 1a,b shows results recorded for the anomalous dispersion-pumped fiber with pump pulse energies of respectively 2 nJ and 5 nJ (coupled into the fiber). The two smaller graphs to the right of Fig. 1b show detail of the fringe pattern of the lower (red dotted box) and higher (blue dotted box) coupled energy. As could be expected for the anomalous dispersion-pumped PCF, strong degradation of the phase coherence can be observed upon increasing of the coupled pulse energy. This corresponds to a situation, when initially (for the lower pulse energy) the fiber length (17 cm) is larger than the characteristic nonlinear lengthscale^33^ (here L_NL_ = 1/γP_0_ = 1.25 cm, where γ is the nonlinear coefficient, P_0_ – peak power) and shorter than the modulation instability lengthscale (L_MI_ = 16L_NL_ = 20 cm). Therefore, the 17 cm long fiber is not long enough for the MI amplification of noise to take place and the SC maintains rather high degree of coherence in the entire spectral width. Upon increasing of the pump pulse energy to 5 nJ, the L_MI_ drops to about 8 cm, consequences of which are immediately visible in Fig. 1b with the coherence lost practically everywhere across the spectrum. Some interference fringes could still be observed red-shifted from the pump wavelength by up to roughly 1720 nm, where the solitons following fission have not yet participated in MI generating noise. Spiky features around 1400 nm and 1900 nm, visible both in the spectrum and in the interferogram in Fig. 1b come from the presence of OH ions in the fiber glass, and should not be mistaken for interference fringes.

Figure 1c–e contain interference measurement results performed for SC generation in the ANDi fiber. The three graphs correspond respectively to the pump pulse energies of 2 nJ, 3 nJ and 5 nJ. By allowing a significant part of the Raman response into the perspective of the pump pulse, the ANDi supercontinuum development becomes dominated with Raman scattering and significant losses of its phase coherence can be expected. Depending on the glass of the fiber (the shape of its Raman response), this detrimental effect can be expected for pump pulse durations longer than 200–300 fs^21^,^24^. The time length of the Raman oscillations, calculated from the measured Raman scattering spectrum of the fiber core glass, is slightly shorter than about 500 fs^24^. Therefore, considering the 390 fs duration of the pump pulse used here, it comes as a surprise that the SC spectrum in the demonstrated ANDi nonlinear fiber remained coherent for all investigated coupled energies. Specifically, the coherence is maintained, even when the spectrum begins to exceed the self-seeded SPM band. In Fig. 1c, the SC is still contained within the SPM-dominated range of wavelengths. In Fig. 1d,e the sidebands of spectrum evolve into wavelengths blue-shifted and red-shifted from the pump. Due to the interaction of the Raman sidebands with FWM, amplification of noise should take place. Instead, the coherence degree remains close to unity. A drop of 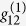 towards 0.7 can be observed at the blue-shifted edge of the SC. This is explained by the fact, that the particular ANDi fiber used, according to linear simulations, becomes few-moded at wavelengths shorter than about 1350 nm. The slight coherence deterioration is then assigned to mode beating. OH absorption can also be noticed at around 1400 nm. The interference fringes are present across the entire width of the SC spectrum, for all the three coupled pump pulse energies. Specifically, fringes can be clearly seen at the red-shifted edge of the spectrum – as shown in the green dotted inset to Fig. 1e, which is where the coherence deterioration of the ANDi spectrum due to Raman scattering could be expected to be the strongest^21^,^31^. Therefore, the decoherence effect due to the few moded core is “contained” to the short-wavelength edge of SC and is not a property affecting the dynamics of SC over its entire wavelength range. For this reason, the excitation of the two-three immediately higher order modes below around 1350 nm of the ANDi SC was not considered in the further analysis.

### Spectral intensity fluctuations and spectral correlation analysis

**Figure 2:**
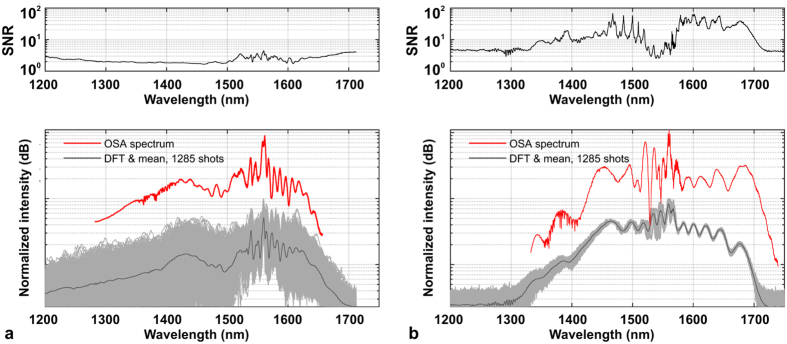
The SNR and the ensembles of single-shot spectra obtained using DFT and averaged spectra recorded with an OSA before the stretching fiber for the investigated soft glass fibers (**a**) anomalous dispersion pumped PCF and (**b**) all-solid, all-normal dispersion PCF. Video recordings showing the shot-to-shot performance of the SC on the oscilloscope and OSA screens in real-time during measurement are included in the supplementary files.

**Figure 3:**
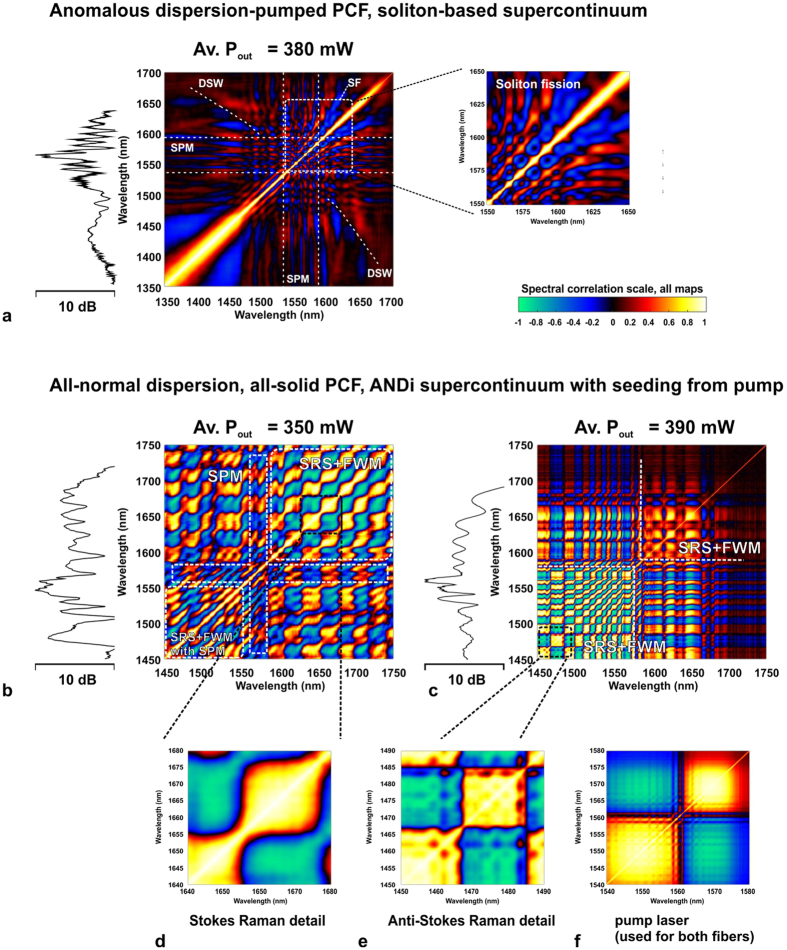
Spectral correlation maps obtained with DFT for investigated soft glass fibers. Typical features of self-phase modulation (SPM), soliton fission (SF) and calculated location of the dispersive wave (DSW) are indicated for the soliton-based supercontinuum. ANDi supercontinuum correlation is shown indicating formation of pump-seeded Raman components, first at Stokes-shifted wavelengths (lower in-coupled pump power^24^), followed by anti-Stokes shifted wavelengths (higher in-coupled pump power), SRS – stimulated Raman scattering, FWM – four-wave mixing. Spectral correlation of the pump laser line is shown in the bottom-right.

Fluctuations of intensity in the SC spectrum were investigated using the DFT approach (see the Methods section for a description of the measurement setup and procedure). Comparison of the shot-to-shot variability of SC spectra in the two discussed nonlinear fibers is shown in Fig. 2. The presented data includes two cases, shown in wavelength scale obtained from the oscilloscope time-base using the time-to-frequency mapping formula (1) – provided in the Methods section – and the signal to noise ratio (SNR), here calculated as the ratio of the mean to the standard deviation of the SC signal. Two video recordings showing the oscilloscope and optical spectrum analyzer (OSA) screens are available in the supplementary material. SC spectra measured before the stretching fiber with an OSA, are shown as red traces for both fibers and confirm the correct time-to-wavelength mapping. The small differences of intensity of the ANDi spectrum at its long-wavelength edge (larger intensity before stretching fiber, Fig. 2b) is assigned to increasing loss of the DCF-38 stretching fiber, which did not influence the shot-to-shot statistics. A dramatic intensity stability advantage of the ANDi spectrum over soliton-based generation is quantitatively evidenced by the experimental data in Fig. 2. In the case of anomalous dispersion-pumped fiber (Fig. 2a) the amplitude of spectral intensity oscillation from shot-to-shot remains over 7 dB across the entire spectrum. This supercontinuum develops in a typical way for the anomalous dispersion and sub-picosecond pump pulses. The primary processes constituting the broadening are self-phase modulation (SPM), followed by SF. Correlation “foot-prints” of these two phenomena are identified in the spectral correlation map in Fig. 3a (refer to Methods on the computation details of the maps in Fig. 3) and are similar to spectral correlation features reported for anomalous dispersion fibers by other groups^14^,^30^. Dispersive waves (DSW) related to the red-shifting solitons are also observed in the spectrum in the area of 1350–1450 nm (Fig. 2a). This assignment is in agreement with theoretical prediction using the relation (3) given in Methods. The corresponding area of correlation map at the intersection of DSW spectral components (1350–1450 nm) and soliton wavelengths (1600–1675 nm) contains an irregular and weakly correlated area along a calculated DSW location curve, together with three, stretched and anti-correlated spots, roughly corresponding with the anti-correlated wavelengths bordering the SF pattern at the long-wavelength edge of map.

The ANDi spectrum is stable to within less than 1 dB for almost its entire extent (Fig. 2b), except for an area immediately next to the pump wavelength, where increased noise is assigned to beating between the SPM and four-wave mixing (FWM) – Raman scattering components^24^. SNR, which here is a measure of the amplitude of the shot-to-shot resolved spectral fluctuation, for the ANDi spectrum is two orders of magnitude higher than in the anomalous dispersion SC. The difference in the stability of both SC spectra can be directly observed also in the video recordings attached as multimedia files.

It can be seen, that in case of the anomalous dispersion-pumped SC, the oscilloscope trace is unstable, indicating dramatically low shot-to-shot stability of spectrum. At the OSA screen this is almost unperceptive, due to the averaging of the measured signal. In the case of the ANDi SC, the temporal profile seen in the oscilloscope screen is stable, and corresponds very well to the averaged optical spectrum measured with the OSA. Shot-to-shot stability of the ANDi spectrum under pumping, which allows a meaningful contribution of Raman scattering into the nonlinear mechanics, was demonstrated in our previous work^24^. We claimed that a part of pump laser light, unconverted by nonlinear processes and propagating in the photonic cladding, continuously seeded the spectral broadening in the core. Here, we show that the development of the ANDi spectrum in these conditions can be divided into two phases, depending on the in-coupled average pump power (power at the output of nonlinear fiber was measured, neglecting loss over a 17 cm long section). At an in-coupled average pump power of 350 mW, the anti-Stokes FWM-Raman components were distorted with SPM (due to beating since this spectral overlap was not phase-matched). The Stokes-shifted FWM-Raman components were clearly distinguishable in the spectral correlation map (Fig. 3d). Indeed, when compared with the experimentally recorded correlation map of the pump laser line (Fig. 3f), a wavelength jitter-like pattern can be observed, multiplied in the red-shifted area of the 350 mW ANDi correlation map^24^. Upon increasing the in-coupled pump power to 390 mW, the spectral correlation pattern of the ANDi fiber (Fig. 3c) at the Stokes wavelengths is smoothed out, which is consistent with FWM-Raman scattering becoming the dominant process in the formation of the spectrum at these wavelengths, at the cost of SPM.

**Figure 4:**
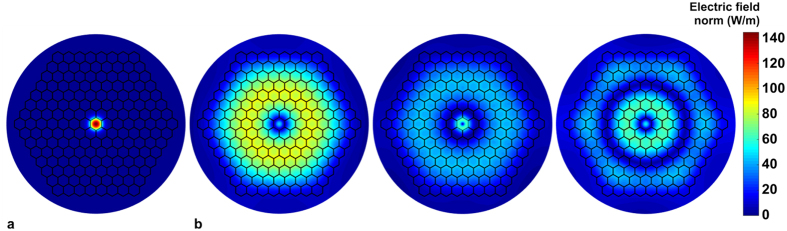
Examples of transverse modes supported by the fiber, obtained using finite element method at 1560 nm, shown in a common color scale (**a**) fundamental mode in the fiber core, (**b**) typical higher order modes overlapping the core from the photonic cladding (see Methods section for details on the fabricated fiber structure).

Passing this “symbolic” threshold of the pump power changes the correlation pattern of the blue-shifted part of ANDi spectrum, as seen in Fig. 3b,c in the correlation maps generated for data recorded at 350 mW and 390 mW of measured output average power. The SPM-induced ripple, present in the 350 mW data, is smoothed out at 390 mW, giving place to a well-defined correlation pattern also carrying the pump laser jitter “foot-print” (Fig. 3e). We note that in previous reports on either the anomalous dispersion-pumped SC or the normal dispersion-pumped SC, Raman scattering was identified as the process contributing to the amplification of noise by modulation instability^21^,^34^. On the contrary, in specific cases, such as the continuous-wave SC generation, Raman scattering-assisted broadening has been credited for an improved noise performance^35^. The results of our experiments suggest, that the contribution of the Raman process to the ANDi SC dynamics in our case can also be related to noise suppression. Numerical simulations are used in the next section to explain this in detail. As means of an introduction to this, we note that the ANDi photonic crystal fiber of the all-solid glass design used in this work (see Methods section), provides the propagation conditions in the photonic cladding with multiple higher order modes overlapping the core. Field distributions of selected higher-order modes with intensity overlap integrals (meant as mode matching, see Methods) between the cladding and core in the range of 3–30% were readily obtained using the finite element simulations. Exemplary mode field distributions are shown in Fig. 4. Due to low nonlinearity in a 35 μm diameter photonic cladding, the fraction of pump light which excites these modes upon coupling into the fiber, does not experience any considerable nonlinear conversion during propagation. This has practical implications. A seed signal (part of the pump pulse propagating in the photonic cladding) experiencing only dispersive stretching without nonlinear distortions nor noise seeded contributions, maintains its phase from shot-to-shot. In other words, it is a deterministic seed signal for the nonlinear process in the PCF core. The seed signal also remains coherent with the pump at all times, automatically fulfilling the condition of at least partial coherence between the seed and the pump for efficient spectral seeding^12^,^14^.

### Simulations of nonlinear propagation

**Figure 5:**
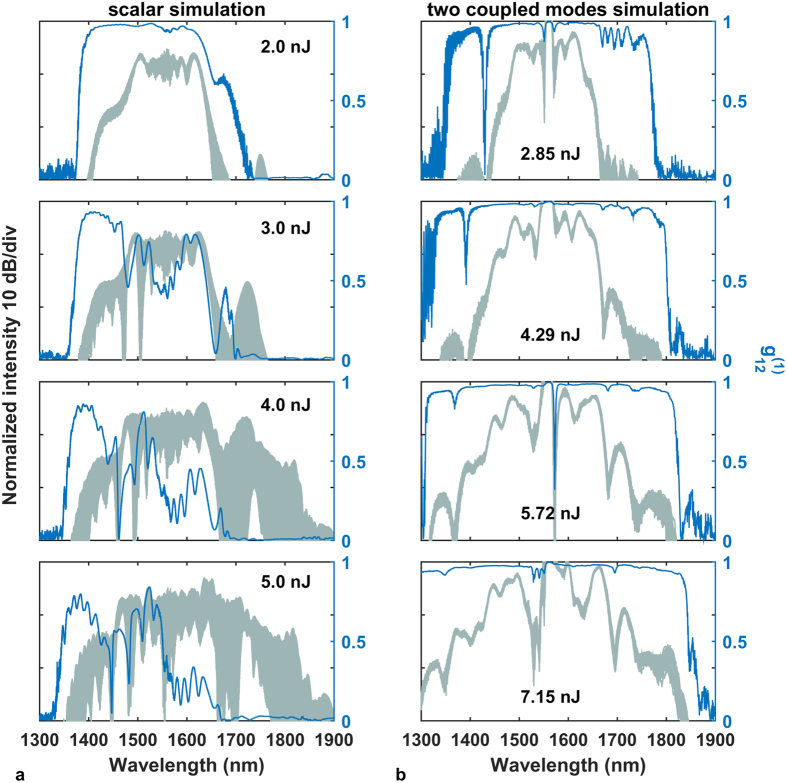
Supercontinuum spectra and first order coherence profiles obtained numerically using (**a**) scalar GNLSE model and (**b**) vector GNLSE with two coupled modes, one representing the SC and the other one representing the seed signal from PCF cladding. Energy values are given for pump pulses coupled into the fiber.

We performed numerical modelling of the formation of the supercontinuum in the ANDi fiber in order to assess theoretically the seeding effect. Prepared models used the generalized nonlinear Schrödinger equation (GNLSE). Modelling of the seeding in our ANDi PCF required a multimode (MM) GNLSE approach^36^,^37^. We used a scalar GNLSE model proposed by Travers *et al.*^38^ as the starting point. GNLSE in that realization is solved in the frequency domain, and can be easily extended to a frequency-domain multi-mode (MM) GNLSE (vector GNLSE) e.g. as proposed by Khakimov *et al.*^39^ (details on nonlinear modelling are included in the Methods section). The noise in the modelling was included as one-photon-per mode^40^. We performed two sets of simulations. In one hypothetical case, we assumed fundamental mode (scalar) propagation, in which the seeding from the cladding modes was not included. In the second simulation, we used a MM GLNSE with the fundamental mode in the core, and one mode from the photonic cladding, with a 10% mode matching (overlap integral, for details see Methods) with the core. Each set of simulations was repeated for a range of pump pulse energies, i.e. 2, 3, 4 and 5 nJ of energy in the core of fiber. A pump pulse energy split between the core and the lattice of 70% and 30% respectively was assumed in the vector simulation (note that this 70/30% energy ratio relates to the split of incident pump pulse energy coupling into the core and into the cladding of the fiber respectively, this is not to be confused with the 10% overlap between the cladding and core modes mentioned earlier). This way, in both simulation sets, the pump pulse energy in the core of fiber was the same (i.e. 2 nJ in the core of scalar simulation, while in the vector simulation there was 2 nJ in the core and 0.85 nJ in the photonic lattice). Both modes in the vector simulation were represented in the model by their dispersion profiles and mode field diameters (roughly 3 μm for the fundamental mode and 35 μm for the cladding mode – see details on nonlinear modelling in the Methods section). We noted that there was very little statistical difference in either scalar or MM simulations for calculations including 250 or 500 shots. Considering this, and the high numerical load of the MM GNLSE simulation, the number of shots considered in this study was limited to 500 for each simulation. Obtained ensembles of spectra and calculated profiles of the degree of coherence 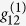 are shown in Fig. 5.

Parameters common to all simulations included the pump pulse shape (Gaussian) and pulse duration (390 fs), the noise characteristics and fiber length (17 cm). A clear improvement of noise performance of the two-mode case over the scalar simulation can be observed with the vector GNLSE generated spectra maintaining a high degree of coherence over all the investigated pump pulse energies. The coherence profile calculated for the scalar simulation (Fig. 5a) drops close to zero at wavelengths red-shifted from pump, which was assigned in our earlier work to Raman scattering dominating the broadening process^24^ and was found consistent with earlier reports of cascaded Raman scattering observed in the UV-violet^31^. The oscillations of 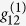 at blue-shifted wavelengths can also be related to anti-Stokes Raman components, which take part in the formation of the spectrum in this area, although their intensity is much lower than that of their Stokes counterparts. The spectrum obtained in the scalar simulation is coherent only for the lowest pump pulse energy, although in such a case the broadening does not exceed the wavelengths covered by SPM, which is less than roughly ±100 nm around the pump wavelength. As soon as the pulse energy becomes large enough for the side lobes of the spectrum to appear, shot-to-shot stability in the scalar simulation deteriorates. This is because the FWM-Raman processes constituting the development of the spectrum outside of the SPM band can pick up from noise, with random phase from shot-to-shot. In the MM-GNLSE case, the fundamental mode in the core is provided with spectral components, the phase of which remains the same from shot-to-shot. These components originate from the photonic cladding mode and are delivered to the core through mode overlap. This way, the nonlinear broadening process in the core is seeded from the cladding with deterministic phase components in every pump laser shot, which keeps the degree of coherence from degrading.

The high degree of coherence obtained numerically from the MM-GNLSE simulation for all investigated pump pulse energies is in a general agreement with the experimental result for the ANDi fiber. A minor exception is that the slight drop of coherence at the blue-shifted edge of spectrum was not reproduced numerically. The reason for this, is that the simulation did not include the effect of excitation of the immediately higher order modes in the core of the physical fiber, which occurs in this wavelength range.

**Figure 6:**
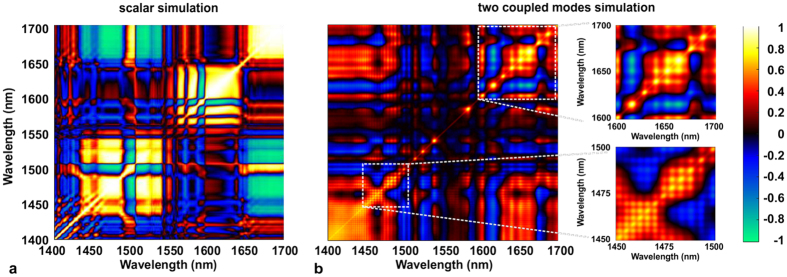
Spectral correlation maps calculated for ensembles of SC spectra generated using (a) scalar GNLSE model and (b) vector GNLSE with two coupled modes (SC and seed).

The spectral correlation maps obtained for both simulations are presented in Fig. 6. The major, highly correlated patterns observed in the map calculated for the ensemble of the scalar spectra (Fig. 6a) were assigned to interaction of FWM and Raman scattering in our earlier work^24^. Spectral correlation in the MM-GNLSE simulation does not contain such features. Instead, a fine grid structure across the entire map can be observed (Fig. 6b). This grid pattern is present in both the correlated and anti-correlated areas, although it is finer at wavelengths blue-shifted from the pump, than it is for the red-shifted wavelengths, as shown in close-ups in Fig. 6b. The map in Fig. 6b is different than the experimental maps in Fig. 3b,c. The presence of the correlations grid pattern is the only, however characteristic, common feature of the MM-GNLSE map and the map obtained experimentally (Fig. 3b,c). This fine grid pattern appearing in the simulated correlation map in Fig. 6b is assigned to FWM-Raman orders, similarly to the case of experimental map (Fig. 3b,c). In the simulation, there is only one higher order mode considered in the cladding, which provides the seed signal to the nonlinear process in the core. At the output of the fiber, for which the map in Fig. 6b was calculated, the seed signal is stretched by dispersion (but not distorted nonlinearly due to large effective area of the cladding) and is available across the entire spectrum of the fundamental mode propagating nonlinearly in the fiber core. At every consecutive pump laser shot, this seed provides the same phase component to the spectral broadening in the core, but it comes from just one cladding higher order mode considered in the simulation. The combination of the single higher order mode origin and full temporal overlap with the core mode at the output of fiber (which is discussed further below) makes the correlation grid pattern fine and regular (across all wavelengths). By comparison, the experimentally obtained correlation patterns (Fig. 3b,c) are a product of an influence of a multitude of higher order modes on the fundamental mode in the core. These modes propagate with different dispersion characteristics and therefore their interaction with the core mode can cover different fragments of spectrum or can take place at different propagation sections along the fiber. The pattern observed in the correlation map is therefore an effective spatiotemporal average of these interactions taken across all the modes, which can be excited in the photonic cladding and which have an overlap with the mode in the core.

The absence of this grid pattern in the scalar GNLSE simulation, supports involvement of the pump signal from the photonic cladding in that the grid pattern does appear in the vector simulation. The improvement of the degree of coherence in the MM GNLSE simulation over the scalar simulation, confirms the assignment of the SC seeding role to the part of pump radiation propagating in the photonic cladding of the PCF.

**Figure 7:**
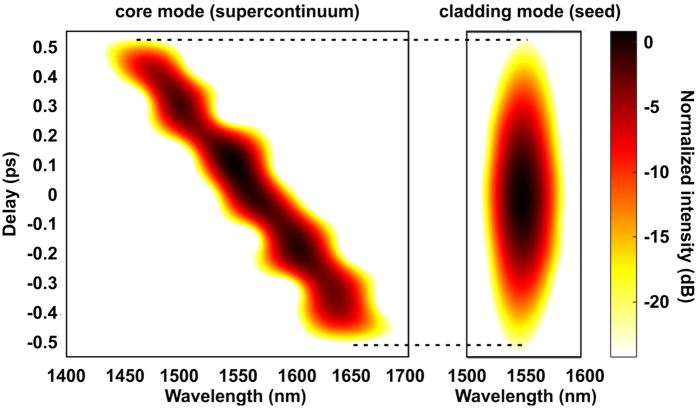
Simulated spectrograms at the output of the ANDi fiber, calculated using MM GNLSE model for the core mode (supercontinuum) and the photonic cladding mode (seed signal).

For the interaction of the seed signal and the SC pulse to take place, both pulses should overlap not only spatially, as discussed earlier, but in the time domain as well. Out of the many possible higher order modes, which can be excited in the photonic cladding of our ANDi PCF, the one included in the MM GNLSE simulation experiences dispersion, which stretches the seed signal over the assumed PCF length comparatively to the stretching of the SC pulse (see Methods for examples of dispersion profiles of modes in the photonic lattice calculated for this fiber). This situation is shown in the numerical spectrograms in Fig. 7, plotted separately for the SC mode from the core and the seed signal mode in the cladding, at the output of the fiber. The temporal overlap between the SC pulse and the cladding radiation in a double-clad ANDi PCF structure, depends on the dispersion profiles of the core and the cladding modes, as well as on the PCF length, and can be used as an optimization parameter in the designing of PCFs for ANDi SC generation seeded directly from the pump laser.

In the vector simulation, just one cladding mode was assumed with full overlap in time and with sufficient overlap in space to uniformly seed development of the supercontinuum in the core. In reality, the mode structure of the fiber’s photonic lattice is very complicated with the different modes having different spatial and temporal overlaps with the core. For example, some modes with stronger confinement around the fiber core could exist in the lattice of the fiber used in the actual experiment. Such modes could experience some nonlinear distortion due to their effective area smaller than that of the other cladding modes, and their overlap with the SC mode would contribute to limiting of the spectral coherence. Present results therefore call for investigation of the spatiotemporal dynamics in such a “double cladding” nonlinear PCF, which would enable optimization of its structure with respect to the process of seeding from the photonic lattice.

## Conclusions

Certain noise sensitive applications necessitate improvement of supercontinuum phase coherence and shot-to-shot intensity repeatability in order to take advantage of the broad bandwidth of such a source, e.g. in the role of a probe beam. Commercially available supercontinuum sources and the majority of new research demonstrators exploit efficient soliton dynamics for spectral broadening, albeit at the cost of large shot-to-shot variability of spectrum. With the use of interference fringe visibility measurements and DFT measurements, we have demonstrated a direct comparison of the phase coherence and the shot-to-shot spectral fluctuations of a soliton-based SC and an SC contained completely in the normal dispersion range of wavelengths of a nonlinear fiber. We have experimentally shown a two-orders of magnitude SNR advantage of the ANDi SC over soliton-based SC for 390 fs long pump pulses. Using spectral correlation maps, obtained from experimental datasets, as well as finite element method numerical simulations of the ANDi fiber mode structure and vector GNLSE propagation simulations, we explained the mechanism of maintained spectral coherence of the ANDi SC, which was maintained despite unfavorable pumping conditions enabling detrimental Raman scattering. The design of the ANDi PCF used here has been discussed earlier for its potential to engineer a flattened, spectrally broad normal dispersion profile^20^. The present results allow us to postulate this all-solid, “double-cladding” structure as a viable alternative for coherence stabilization in ANDi SC generation driven by the available, high-power sub-picosecond fiber-based pump lasers. With this, our results are of practical value for the community of device engineers, as well as users, since they demonstrate the feasibility for a supercontinuum source combining true shot-to-shot repeatability of generated spectrum, with a small foot-print of the entire, high output power device setup.

## Methods

### Experimental setup

**Figure 8:**
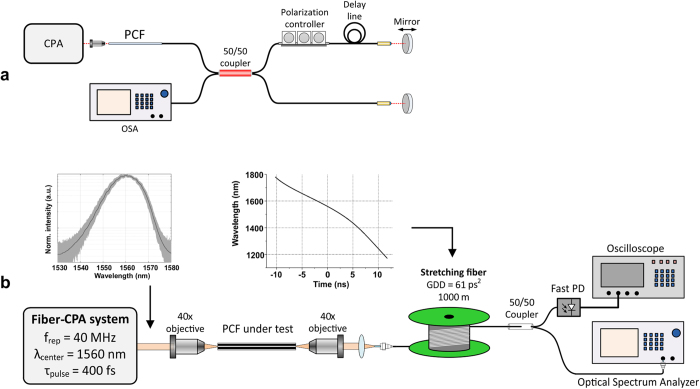
Experimental setups used in this work (**a**) fiber-based Michelson-type, unequal path-length interferometer for phase coherence measurements, (**b**) dispersive Fourier transformation setup, the shot-to-shot resolved spectrum of the pump laser emission and the time-to-wavelength mapping relation of the DCF-38 stretching fiber.

The experimental setups for measuring the phase coherence and the shot-to-shot resolved spectra of SC generated in the PCFs is depicted in Fig. 8. The tested PCFs are pumped by a fiber-based chirped pulse amplification (CPA) system, which delivers 390 fs-short pulses centered at 1560 nm with 40 MHz repetition rate. The design of the CPA was described in detail in previously published work^24^. The pulses from the pump laser are launched into the PCF through a microscope objective with 40× magnification. The maximum incident average pump power used in the experiments was about 1.1 W. By measuring the average pump power at the output of the studied PCFs, we estimated the coupling efficiency at about 30%.

The phase coherence of the supercontinuum was verified in a Michelson-type fiber interferometer with unequal path lengths. The setup of the interferometer is depicted in Fig. 8a. The generated SC is coupled into a single-mode fiber and divided into two parts via a 50% coupler. One of the arms contains a fiber-based polarization controller, in order to adjust the polarization and to achieve the maximal interference signal. To allow fine tuning of the path difference, the mirror in one of the arms was placed on a translation stage. The interferograms were recorded using optical spectrum analyzer (Yokogawa AQ6375) with 0.2 nm resolution. The DFT setup is shown in Fig. 8b. The generated SC is first coupled into a 1000 m long stretching fiber, Corning Vascade S1000 (DCF-38), with total group delay dispersion of 61 ps^2^. The laser line has also been measured using this setup. In order to resolve it spectrally, a longer section of stretching fiber of 3700 m was used. The coupling efficiency to the stretching fiber was intentionally decreased to a few percent, in order to avoid nonlinear spectral broadening at this stage. Afterwards, the signal was divided into two parts. One part was detected by a fast photodiode with 16 GHz bandwidth (Discovery Semiconductors DSC2-50S) and observed on an oscilloscope (Agilent Infiniium DSO91304A) with 13 GHz bandwidth. Spectral resolution after mapping of the oscilloscope time-base was 2.1 nm, and it was limited the oscilloscope’s bandwidth. Vertical resolution (dynamic range) of the DFT trace before normalization was 20 dB. The second part of the signal was observed on the optical spectrum analyzer (again Yokogawa AQ6375). The length of both PCFs was 17 cm, which was enough for generating a spectrum fitting into our DFT setup and was also convenient for handling.

### Methods of analysis

The modulus of the complex degree of coherence was calculated from the contrast between the interference fringes, according to 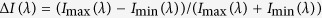, where I_max_ and I_min_ are respectively the envelope and the baseline of the interference fringe pattern. Before calculating of the fringe contrast for the case shown in Fig. 1b, the OH absorption peaks at 1350–1400 nm and 1800–1950 nm were removed from the interferogram, by applying a median filter in these two wavelength ranges. This did not distort the information on the coherence, because the measured spectrum and the interferogram in Fig. 1b were almost indistinguishable in the area of the OH absorption bands (any interference in the data shown in Fig. 1b was limited to the 1400–1800 nm wavelengths). In the DFT measurements, the SC spectra were monitored for spectral distortions before and after the stretching fiber using an OSA. The oscilloscope enabled recording of a trace which fitted around 256 consecutive shots and several traces were recorded for each fiber, hence over 1200 shots were used in the analysis. Oscilloscope time-base was converted to frequency (and to wavelengths) as discussed in^29^,^30^, taking into account higher order dispersion terms of the stretching fiber:


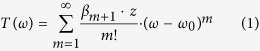


Dispersion terms up to β_7_ were included in the calculation. Inclusion of dispersion terms higher than β_7_ did not change the mapping relation in any meaningful way. The stretching fiber’s dispersion has been measured for purpose of β_i_ calculation in the range of 1250–1820 nm, and the time-to-wavelength mapping relation obtained using formula (1) is shown in the inset in Fig. 8. Recorded oscillograms were wrapped using a simple computer algorithm and the common time scale was then converted to wavelengths, yielding shot-to-shot resolved SC or pump laser spectra. This experimental data enabled calculation of spectral correlation maps, defined as:


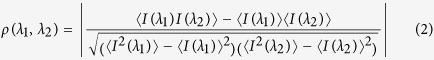


where brackets denote mean over an ensemble of spectra or products of spectra, respectively. For calculation of the maps shown in Fig. 3, full data sets of over 1200 shots were used. In practice, the contrast among the map features was changing only negligibly, when at least 500 shots were used for computation. This was also verified in case of the numerical simulations and numerical maps shown in Fig. 6. Here also the map detail was changing imperceptibly for larger shot counts, hence the final results shown in Fig. 6 were based on a 500 shot simulation in order to limit the computation time of the numerical results.

Location of the dispersive waves for the anomalous dispersion fiber has been calculated using the formula^41^:


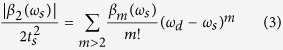


where ω_s_ and ω_d_ are soliton and DSW angular frequencies, while t_s_ = t_0_/(2N–1) is the soliton duration (with N denoting the soliton order).

### Fibers

**Figure 9:**
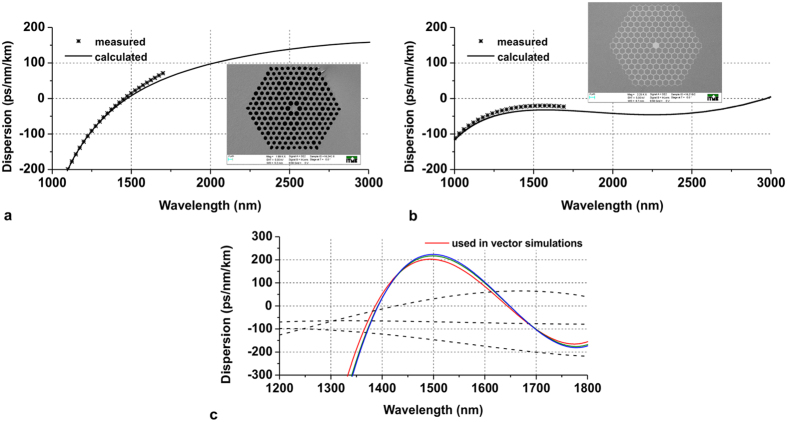
Photonic crystal fibers designed and fabricated at ITME, used for this study (**a**) lead-bismuth-gallate, air-glass PCF, with zero-dispersion wavelength at about 1500 nm, photonic cladding and core diameters: 40 μm and 3 μm, (**b**) boron-silicate/F2 all-solid glass PCF with all-normal flattened dispersion, photonic cladding and core diameters 35 μm and 3 μm, (**c**) exemplary higher order mode dispersion profiles in the photonic cladding of the all-solid glass PCF, obtained numerically using linear simulations.

Noise performance in the soliton-based type of SC generation was demonstrated in a hexagonal lattice air-hole PCF, with a zero-dispersion wavelength located at about 1500 nm. The dispersion characteristic and image of the lattice are shown in Fig. 9a. The fiber has been designed and fabricated in-house using the stack and draw method. Highly nonlinear, in-house made lead-bismuth-gallate glass was used^42^,^43^. The PCF has been shown to deliver octave or two-octave spanning spectra up to 2900 nm under pumping with 1550 nm pulses and with femtosecond^42^ or sub-picosecond pulse durations^43^. The ANDi PCF, shown with its dispersion profile and lattice layout in Fig. 9b, features an all-solid, hexagonal structure made of an in-house boron-silicate glass and lead-silicate F2 glass from Schott. This fiber has also been designed and fabricated at the Institute of Electronic Materials Technology (ITME) using the stack-and-draw method^20^,^24^. The boron-silicate glass has been designed to have thermal expansion coefficient and rheological parameters similar to the F2 glass, while at the same time refractive index contrast between the two glasses would enable design of an index-guiding structure. The refractive indices of the two glasses, measured at 1550 nm are n = 1.594874 and n = 1.511304 for F2 and the boron-silicate glass, respectively. The core and lattice of the fiber is made of F2 glass, while the glass inclusions in the lattice and the tube around the photonic cladding are made of the boron-silicate glass. Such a structure enables index-guided propagation both in the core and in the photonic lattice, enabling the spectral seeding mechanism of SC generation discussed in the paper.

### Nonlinear propagation model

The vector model used in this work solves equation (4) 39 using the split-step algorithm. It was constructed using the scalar model^38^ as starting point, with the addition of the mode coupling terms 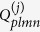 defined with (5) and obtained from linear modelling of the fiber’s mode structure. The complete set of the complex values of 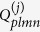 includes 32 terms, which are all possible permutations of p,l,m,n = (1,2). These terms should not be confused with the mode overlap integral (6), where E_1,2_ are complex electric fields in the transverse plane of the fiber, over which the integration takes place. Its value of 0.1 (10%) was given here as reference only. The parameters ε_0_, n_0_, n_2_ and c stand for respectively: vacuum permittivity, linear and the nonlinear refractive indices of the fiber glass, and the speed of light in vacuum. Values of n_2_ were measured at a wavelength of 1240 nm^44^ for the two glasses used for fiber stacking and are: n_2_ = 1.1 × 10^−20 ^m^2^/W for the boron silicate glass filling the inclusions of the photonic lattice and n_2_ = 3.0 × 10^−20 ^m^2^/W for the fiber glass forming the core and the lattice. Because of limited spectral width of the simulation, and with simplicity in mind, the frequency dependence of the mode area was neglected, and fixed values of 6.5 μm^2^ and 212 μm^2^ were used for the core and the photonic cladding, respectively. Core glass n_2_ was taken for the fundamental mode, while the lower value of n_2_ for the glass filling the lattice was used to model nonlinearity of the cladding mode. Values of the effective mode areas, electric field intensities and dispersion profiles were obtained using finite element modelling of the real fiber structure. Specifically, the propagation constant and the inverse of the group velocity for the core mode were β_0_ = 6283104.91 1/m and β_1_ = 1.35 × 10^−9 ^s/m, while for the cladding mode β_0_ = 6118736.21 1/m and β_1_ = 1.71 × 10^−9 ^s/m. The dispersion profiles calculated for the fundamental mode in the core and of several higher order modes in the lattice are shown in Fig. 9b,c. Most of the modes in the photonic lattice had dispersion profiles similar to the ones drawn with solid lines in Fig. 9c and the cladding mode used in the MM-GNLSE simulations was represented with the dispersion profile marked with the red solid trace. Subscript (p) in the symbols representing the propagation constant β_0_, the inverse of the group velocity β_1_ and the dispersion terms β_n_, indicates values for the cladding mode. The Raman response was modelled after Blow and Wood^45^ with the parameters f_R_ = 0.2 (Raman contribution to Kerr nonlinearity), τ_1_ = 5.5 fs and τ_2_ = 32 fs most closely resembling a Lorentzian fit of the measured Raman scattering spectrum of the fiber’s core glass. Furthermore, in the model (4), the symbols 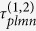 stand for the timescale of the optical shockwave formation and N_p_ represents normalization coefficients.


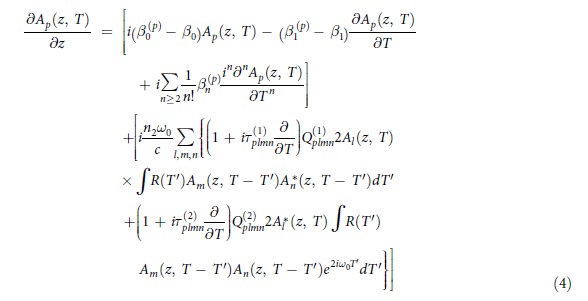



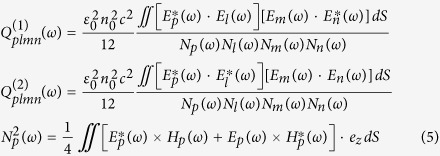



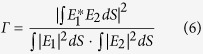


Finally, the modulus of the complex first order degree of coherence has been calculated from the simulated ensembles of supercontinuum pulses, using the formula^10^,^33^:


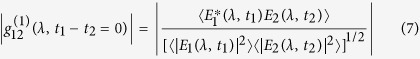


where angle brackets <,> stand for an ensemble average taken over the pairs of the simulated SC pulses.

## Additional Information

**How to cite this article**: Klimczak, M. *et al.* Direct comparison of shot-to-shot noise performance of all normal dispersion and anomalous dispersion supercontinuum pumped with sub-picosecond pulse fiber-based laser. *Sci. Rep.* **6**, 19284; doi: 10.1038/srep19284 (2016).

## Supplementary Material

Supplementary Information

Supplementary video 1

Supplementary video 2
