# Supplementary material for: Direct comparison of shot-to-shot noise performance of all normal dispersion and anomalous dispersion supercontinuum pumped with sub-picosecond pulse fiber-based laser
Source: Sci Rep. 2016 Jan 13;6:19284. doi: 10.1038/srep19284 (PMC4725351; doi:10.1038/srep19284)
Supplement: Supplementary Information [file srep19284-s1.doc]

**Direct comparison of shot-to-shot noise performance of all normal dispersion and anomalous dispersion supercontinuum pumped with sub-picosecond pulse fiber-based laser**

Mariusz Klimczak,^1*^ Grzegorz Soboń,^2^ Rafał Kasztelanic,^1^ Krzysztof M. Abramski^2^ and Ryszard Buczyński^1,3^

**^1^ Glass Department, Institute of Electronic Materials Technology, Wolczynska 133, 01-919 Warsaw, Poland**

**^2^ Laser & Fiber Electronics Group, Wroclaw University of Technology, Wybrzeze Wyspianskiego 27, 50-370 Wroclaw, Poland**

**^3^ Faculty of Physics, University of Warsaw, Pasteura 7, 02-093 Warsaw, Poland**

* Correspondence and requests for materials should be addressed to M.K. (mariusz.klimczak@itme.edu.pl)

Supplementary information includes two video files: “Supplementary_video_1.avi” and “Supplementary_video_2”.

**Supplementary video 1**

The file “Supplementary_video_1.avi” contains a video recording of shot-to-shot resolved spectral fluctuations of supercontinuum recorded in the anomalous dispersion-pumped fiber. The upper instrument is an optical spectrum analyzer and it shows the averaged spectrum measured in real-time at the output of the photonic crystal fiber. The lower instrument is an oscilloscope, showing in real-time the supercontinuum pulse spectra, resolved shot-to-shot in the dispersive Fourier transformation setup, measured by a fast photodiode detector at the output of the stretching fiber.

**Supplementary video 2**

The file “Supplementary_video_1.avi” contains a video recording of shot-to-shot resolved spectral fluctuations of supercontinuum recorded in the all-normal dispersion (ANDi) fiber. The upper instrument is an optical spectrum analyzer and it shows the averaged spectrum measured in real-time at the output of the ANDi photonic crystal fiber. The lower instrument is an oscilloscope, showing in real-time the supercontinuum pulse spectra, resolved shot-to-shot in the dispersive Fourier transformation setup, measured by a fast photodiode detector at the output of the stretching fiber.
